# Misrepresentations

**Published:** 1882-05

**Authors:** Ad. Lippe

**Affiliations:** Philadelphia


### MISREPRESENTATIONS.

AD. LIPPE, M. D., PHILADELPHIA.

Another Dead Sea fruit has ripened, and has fallen from that famous tree of freedom planted under the auspices and acclamations of the pseudo-homœopaths—alias eclectics, alias Thomsonians, on the 8th day of June, 1870, at Chicago. The fruit, fully ripened, and fully dressed, sugared and spiced, has been served up to the homœopathic profession ostensibly, but in reality to all sorts of medical men, in the March number of the New York *Medical Times*, page 362.

MISREPRESENTATION No. 1. That the homœopathic profession is actually divided between high and low-potency men. It is no doubt the design of the author of this paper to create such a division. He declares that the theory of dynamization is rickety, mischievous and erroneous; worthless chaff; a medical illusion which will be remembered only with jesting and mockery. That the work of elimination must go forward, and that the reports of nondescript dynamic cases must be stamped as "*lies*." To accomplish this end it is proposed to *remove* all public teachers in our colleges who represent these obnoxious doctrines. Our state and local societies are urged to declare that all practice with potencies higher than the twelfth be classed as dynamic. This is the Dead Sea fruit presented to a profession by a set of men who style themselves "liberals." The real difference between the allopathic and homœopathic school of medicine is, and consists of, the declaration of the allopathists that diseases have a material origin, and that organic bodies are governed by the same laws governing inorganic bodies, that therefore, material and chemical means are necessary to remove disease. While the homœopathists, declared from the beginning of the promulgation of their school that diseases have not a material origin, but an immaterial (dynamic) origin, that organic bodies were not governed by the same laws governing inorganic bodies, that therefore, under pressure of ordinary logic, diseases must not be treated by material and chemical means, but that disease having an immaterial (dynamic) origin could only be treated by immaterial (dynamic) means.

The paper under consideration declares that there exists a low-potency party "*who acknowledge, to the fullest extent, that they are ardent and thorough disbelievers in the ridiculous theory of dynamization.*" Here is clearly expressed the point of dissension between the true and the fictitious Homœopathy. Homœopathy without the theory of immaterial (dynamic) causes of diseases, and the logical necessity to combat such disease by immaterial (dynamic) remedies would be just like the play of Hamlet with Hamlet left out. A person who considers the theory of dynamization a fanciful creation of Hahnemann testifies against himself, if he proclaims such absurdities. He shows his utter ignorance of all of Hahnemann's teachings; he shows that he has not followed the great philosopher in his strict inductive method; he proclaims himself to be a thorough disbeliever in Homœopathy, and his unbelief becomes more apparent when he denies the immutableness of the Law of the Similars, on which the whole structure of Hahnemann's inductive method rests. He proclaims himself to be an eclectic to all intents and purposes, and the division between high and low-potency men appears to be a miserable subterfuge, a false issue. It is more than absurd to limit the homœopathic school to the 12th potency; if there is any medicinal virtue in the 12th potency, why not in the 12 millionth potency? These liberal eclectics attempt just this absurdity, and the only excuse that can be offered in their behalf is that they are deplorably ignorant of the principles of Homœopathy.

Misrepresentation No. 2.—In conclusion, the paper states: "The high-potency party have held sway too long. * * * This hypothetical method of practice has had its ephemeral existence as chiefest of medical illusions!" * * *

In the first place, there does not and never did exist a high-potency party as such, or under any such name. There did, does and will forever, exist an honest, conscientious set of men who practice the healing-art as promulgated by its founder; many of them have had recourse to high potencies; but when and where, by word of mouth as public teachers or as writers, did any one of the many high-potency men denounce or condemn those who consistently cured the sick as best they could with low potencies? When and where have any of these men been guilty of declaring cures with low potencies no cures at all, or demanded them to be considered as lies—when and where, you men so ingenious in your misrepresentations, did any one of them so far forget himself? The ephemeral existence of these men is at an end. You forget your own dignity if you insist on giving these men the lie direct—you *misrepresent*, and why? You have no other argument left you. The tub on which you tried to stand lost its bottom when you so cringingly expressed your longing for recognition by a school which you deceived by your misrepresentations, and who inflicted upon you the well-deserved punishment of recognition which, did you know history, is equivalent to "*annihilation!*"

The homœopathic school *is* divided, and you, speaking professedly for a majority, have thrown down the gauntlet! Your assertions being misrepresentations, it is to be hoped that nobody will pick up your gauntlet. How much better will it be if you are left to your chosen misrepresentations and with your joys over the cunningly-assumed liberality of the common school of medicine. With your exultations over their "recognition" you are punished sufficiently already.

The homœopathic school is divided; we have a steadily-increasing number of consistent homœopathicians who follow the teachings of the founder of our healing-art; we have men who, sailing under false colors, profess to be homœopaths, but in reality practice eclecticism. Let us now indulge in a short retrospect that we may fully appreciate the situation. In 1844 the first session of the American Institute was held; there were present sixteen members, and the posological question did not divide them; they were all and every one of them homœopathicians in the fullest sense of the word; their great aim was to develop the healing-art by following the great master in augmenting our materia medica, and thereby add to our means of applying our law of cure. These few earnest men were the early pioneers of our school, strictly adhering to the teachings of the master, and having no other aim before them than the vindication of our healing-art. Showing by their successes the great superiority of the new over the old school, they did fearlessly combat error and prejudice. The number of these self-sacrificing pioneers gradually increased, and they did conquer all the various obstacles they encountered, through their fidelity to principles. It was owing to the great sacrifices of these men that Homœopathy was firmly established in this country. The least possible deviation from the strictest methods of our school was out of the question; it was not thought of; one and all of these pioneers fully testified to the correctness of the teachings of the master, and the evidence brought to their mind was their success. Had these pioneers not shown these great and convincing successes, our healing-art would have been extinguished then. These pioneers inspired others with the same spirit of persistently following the master's teaching, and the same men have by the same means, and by the same successes, established our healing-art firmly, and it is now, in these days, utterly impossible to persuade the men who were true to their principles that a new, more liberal practice, denying some of the master's teachings and again resorting to long-abandoned palliative means, could ever take the place of, or be amalgamated with, the faithful practice of the true followers of the master. These consistent men know that truth and error cannot possibly exist together; they demand (not cringingly beg for) recognition by the allopathic school, the recognition of the law of cure, of the single remedy and also of the minimum dose;* not singly but collectively, and they are strengthened in their faith by the discoveries in sciences and arts confirming all the teachings of its master, as well as by the gradual but certain recognition by the degenerating old school of one after the other of the principles governing our healing-art. The old school, they contend, must come to us and must surrender unconditionally; they ask no favors.

At about the same time, when the homœopathists united themselves, and formed an organized society, the American Institute of Homœopathy, the Thomsonians, alias herb doctors, had made great inroads on the medical practice in the State of New York especially; they had been bitterly persecuted by the allopathists, and gained adherents under this mode of opposition. Gaining very considerable influence, they abandoned their original declarations of only using

*The minimum dose is the dose just sufficient to cure, and has so been demonstrated always. The ignorance of the writer of the paper we alluded to construes it as meaning "the *highest* potency," a willful, ingenious and malicious misrepresentation.

vegetable medicines, and resorted clandestinely to the prevailing abuse of mercurial preparations, when in their individual judgment it appeared best. Persecution had helped them very much, and some astute members of the New York State Society proposed, and finally carried out their design, of annihilating their rivals by suddenly assuming great liberality. All oppressive measures were at once removed, recognition was granted them, and the remedy was successful. Thomsonianism became extinct, and while recognition had worked annihilation, these ex-Thomsonians formed a new sect under the title of eclectics. They were allowed to establish their own colleges and societies without hindrance; the allopathists allowed them full freedom of medical opinion and action. Guided by no principles, they very soon fell into discredit and without any persecution by the allopathists, the people discovered their shortcomings, they violated their chartered rights, flooded the country with illegal diplomas; the press exposed them, and they were finally and disgracefully annihilated. In the meantime, some of them, fully aware of the impending catastrophe, were seeking shelter, and became professing homœopaths, to reap the fruits of the labors of the pioneers in our school. They joined our societies, and it was claimed by some good and honest men that we should accept them in full membership, and give them freedom of medical opinion and action, by which means, it was argued, they would come to accept and return to that purity of practice we all so much desired to be established. They came in large numbers, and under the pretext of augmenting our materia medica, we find them the publishers of New Remedies, mostly taken from the former text-books of the Thomsonians. Growing bolder as their numbers steadily increased, they abandoned gradually the practice of the pioneers, who, by their faithful adherence to the teachings of the master, had fully established confidence in Homœopathy. We find them first alternaters, then resorting to crude drugs, later returning to palliative treatment, and lastly they claimed the right to use any means they chose to find best, according to their own individual judgment; resorting to misrepresentations and ridicule of the only friends they had found when they stood alone and under fears of the just law, which was called to aid the profession to rid itself of a set of men who had been guilty of the violation of the laws of the land. Under such misrepresentations as we have exposed in this paper, the allopathic school of medicine was deceived, and believing that these men represented the school of medicine called Homœopathy, the old and so successful remedy was once more applied; the allopathists suddenly became generously "*liberal*," and the allopathic Medical Society of the State of New York passed a resolution of "*recognition!*" The only comment we make at present is that history repeats itself.

MISREPRESENTATION No. 3.—In the March number (1882) of the *American Observer*, we find on pp. 145 and 146 another gross and malicious *misrepresentation!* "The standard of homœopathic preparations was erected by Hahnemann. The utmost limit of drug attenuation he placed at the decillionth potency or tenth centesimal dilution. Beyond this he expressly forbade the attenuation of drugs."

Will the author of this bold assertion let the profession at large know *where* in Hahnemann's writings he finds this positive prohibition against attenuating medicines beyond the 30th potency? Does this misrepresenter follow all the injunctions Hahnemann gave us, faithfully? Does he know anything about them? Will he please take up the 5th volume of the *Chronic Diseases*, by Samuel Hahnemann, published in 1839, and will he read the preface to "Arsenic," the very last paper he ever published? He will find on page 496 just the reverse of what he claims Hahnemann said. Here it is, "The true healer must be at liberty to apply the many remedies which nature presents for the cure of the sick just in such quantities, may this quantity *be ever so small* or large, and his experience and experiments must guide him in the use of them for the purpose of curing, in such a form as investigation and experience have taught him to be most useful." As an historical fact we state that Hahnemann, himself, after writing this above sentence, very generally administered much higher potencies than the 30th. How can any one suppose that this great philosopher should ever have thought of limiting the dose? We have exposed two gentlemen guilty of misrepresentations; the one desires the limit to be the 12th, the other the 30th potency; both arbitrarily demand that our societies fix the limits of the dose. There is a more rational way to proceed, and that is this, let these dose fixers relate cases—how they made the experiment, and how the one found by the only possible test, the experiment, that beyond the 12th potency there is no curative powers; while the other proves to the contrary that up to the 30th the curative powers continue, but cease right then and there. *Vide* also Hahnemann's *Organon*, paragraph 247. (The smallest doses).

Again the writer, on p. 145, desires to misrepresent the "Internationals" as wearing the follies of the "crank of the nosode," like a glove in their helmet. Where did this learned writer find the Internationals indorse any crank of the nosode? Where is his logic? If there are six or more cranks among the homœopathic physicians who habitually indulge in misrepresentations, does it thereby follow that all homœopathists are given to this odious habit? Or does it follow that while one compiler or author of the "Transactions of the World's Homœopathic Convention" does in vol. I, p. 801, and in other places, falsify facts, is guilty of flagrant misrepresentations, that therefore all the compilers of the transactions of the American Institute are guilty of misrepresentations? Certainly *not.*

If H. W. T. does not misrepresent himself we hope he will boldly offer resolutions at the next meeting of the American Institute *to officially discountenance the nosode crank and the dynamizationist.* We can spare the nosode crank, but we can't well spare Hahnemann and *his* dynamization; not *we* as homœopathicians, but the nosode crank and the eclectics can well do without him hereafter as they have done heretofore.

